# Responses of Fungal Assembly and Co-Occurrence Network of Rhizosphere Soil to *Amaranthus palmeri* Invasion in Northern China

**DOI:** 10.3390/jof9050509

**Published:** 2023-04-25

**Authors:** Mei Zhang, Kefan Wang, Cong Shi, Xueying Li, Zhenlu Qiu, Fuchen Shi

**Affiliations:** 1 Department of Plant Biology and Ecology, College of Life Sciences, Nankai University, Tianjin 300071, China; zhangmeinku@163.com (M.Z.); kfwang3333@163.com (K.W.); lixueying0206@163.com (X.L.); 1120190471@mail.nankai.edu.cn (Z.Q.); 2 School of Environmental Science and Engineering, Tiangong University, Tianjin 300387, China; shicong@tiangong.edu.cn

**Keywords:** *Amaranthus palmeri* invasion, rhizosphere soil, fungal community, LEfSe, co-occurrence network, keystone taxa

## Abstract

The interaction between invasive plants and soil microbial communities is critical for plant establishment. However, little is known about the assembly and co-occurrence patterns of fungal communities in the rhizosphere soil of *Amaranthus palmeri*. The soil fungal communities and co-occurrence networks were investigated in 22 invaded patches and 22 native patches using high-throughput Illumina sequencing. Despite having little effect on alpha diversity, plant invasion significantly altered the composition of the soil fungal community (ANOSIM, *p* < 0.05). Fungal taxa associated with plant invasion were identified using linear discriminant analysis effect size (LEfSe). In the rhizosphere soil of *A. palmeri*, Basidiomycota was significantly enriched, while Ascomycota and Glomeromycota were significantly reduced when compared to native plants. At the genus level, the invasion of *A. palmeri* dramatically increased the abundance of beneficial fungi and potential antagonists such as *Dioszegia*, *Tilletiopsis*, *Colacogloea*, and *Chaetomium*, while it significantly decreased the abundance of pathogenic fungi such as *Alternaria* and *Phaeosphaeria*. Plant invasion reduced the average degree and average path length, and increased the modularity value, resulting in a less complex but more effective and stable network. Our findings improved the knowledge of the soil fungal communities, network co-occurrence patterns, and keystone taxa in *A. palmeri*-invaded ecosystems.

## 1. Introduction

Alien plant invasion is a significant component of current global change and one of the primary causes of biodiversity loss [1,2]. The successful invasion of alien plants is directly correlated with the alteration of the underground microenvironment, including the alterations in soil characteristics and the composition and function of the soil microbial community [3,4]. All plants have distinct microbial communities, and the population establishment of exotic plants is accompanied by a shift in microbial communities to obtain different nutrient pools, thus bringing growth advantages to invasive plants [5]. Soil fungi are an essential component of the soil microbial community. Fungi can act as mutualists in the synthesis and decomposition of soil organic matter, and they can also prevent plants from establishing as pathogens [6,7,8]. The interaction between plants and fungi is frequently one of the main determinants of plant invasion. There have been extensive attentions on the evaluation of how soil fungal communities react to the invasion of exotic plants [6]. The enrichment of arbuscular mycorrhizal fungi (AMF) species such as *Paraglomus* sp. in soil was found to be one of the mechanisms contributing to the successful invasion of *Chromolaena odorata* [9]. Greater diversity and higher abundance of specific types of fungal communities in invaded soils were associated with the growth and reproduction of invaders [10]. Bamboo invasion increased the alpha diversity, while it decreased the abundance of soil fungal communities [11]. The invasion of *Bromus diandrus* and *Avena fatua* may result in a reduction of multiple soil symbionts that native species rely on for pathogen defense and improved access to soil resources [12]. Co-invasion of pine and their ectomycorrhizal fungi caused a dramatic loss of fungal diversity which could inhibit the recovery and restoration of invaded ecosystems [13].

Molecular ecological network (MEN) analysis based on random matrix theory (RMT) is a potent method for examining the intricate relationships between microbes. In recent years, network analysis has been applied to investigate the co-occurrence patterns of microorganisms in a variety of environments, including rivers, wetlands, lakes, and soils [14,15,16,17]. The microbial groups are positively or negatively linked, exhibiting cooperative or competitive characteristics [18]. Network analysis can also be used to identify keystone taxa, which are highly connected to other species in the network and may have a considerable impact on the entire microbial community [19,20,21]. Keystone taxa, regardless of abundance, have been shown to play an over-proportional role of functional explanatory power in the co-occurrence networks [22,23]. Thus, in order to predict the impact of exotic plant invasion on ecosystem function, it is necessary to conduct a more thorough investigation of the influence of *A. palmeri* invasion on fungal community networks and keystone taxa.

Palmer amaranth (*Amaranthus palmeri* S. Watson abbreviated as *A. palmeri*) is native to the western United States and northern Mexico. It is a dangerous “super weed” that is resistant to many different herbicides [24,25]. The presence of *A. palmeri* will result in significant yield losses if not fully controlled, and it has been regarded as the most agronomically challenging species in the United States [26,27]. In recent years, *A. palmeri* has spread rapidly throughout the Beijing-Tianjin-Hebei region of northern China, displacing native plants and endangering local biodiversity [28]. Previous studies have already indicated that *A. palmeri* invasion enhanced functional traits (i.e., leaf number, plant height, and total biomass) [29], altered soil chemical and biological properties (e.g., total carbon, ammonium nitrogen, and soil extracellular enzyme activities) [30], and influenced bacterial composition and co-occurrence patterns [31]. It is unknown, nevertheless, how the invasion of *A. palmeri* would affect the diversity, composition, and ecological network of the soil fungal community. The purpose of this study was to (1) investigate the composition and co-occurrence patterns of soil fungal communities and (2) compare the significant species and keystone taxa in invasive and native rhizosphere soils.

## 2. Materials and Methods

### 2.1. Sampling Site and Soil Collection

In the Beijing-Tianjin-Hebei region of China, 22 sampling sites (38.74°–40.04° N, 116.36°–117.85° E) were established in 2021 (general information, site description, and vegetation characteristics were shown in Table 1 and Table S1; the map of the sampling sites was shown in Figure S1), in which 5 invaded plots and 5 paired native plots were created in each site to collect the rhizosphere soils of native and invasive plants, respectively. Plant roots, litter, stones, and impurities visible in the samples were taken out, kept at a low temperature, and then transported back to the laboratory. The soil samples were divided into three parts after screening, with one placed at −20 °C for DNA extraction and the other at −4 °C for the content of ammonium nitrogen (AN) and nitrate nitrogen (NN) measurement. The remaining soil samples were dried naturally and used for the determination of soil pH, total carbon (TC), total nitrogen (TN), total phosphorus (TP), and available phosphorus (AP) content. Our earlier investigations provided comprehensive descriptions of the study locations, sample collection, and measurement of physicochemical properties [30].

### 2.2. DNA Extraction, PCR, and High-Throughput Illumina Sequencing

DNA was extracted from soil samples using a Power Soil DNA extraction kit (Mo Bio Laboratories, Carlsbad, CA, USA) according to the manufacturer’s instructions, and DNA concentration was measured using a Thermo Nanodrop 2000 instrument. The fungal primer pair ITS5-1737F and ITS2-2043R with a specific barcode was used to amplify the ITS1 regions of the fungal ITS rRNA genes [32]. The PCR reaction contained 25 μL 2× Premix Taq (Takara Biotechnology, Dalian Co., Ltd., Dalian, China), 1 μL of each primer (10 μM), and a 3 μL DNA (20 ng/μL) template in a volume of 50 μL. The PCR amplification included initialization at 94 °C for 5 min, followed by 30 cycles of 94 °C for 30 s, 52 °C for 30 s, and 72 °C for 30 s, and a final elongation at 72 °C for 10 min. NEBNext^®^ Ultra™ II DNA Library Prep Kit for Illumina^®^ (New England Biolabs, Ipswich, MA, USA) was used for library construction [33]. Illumina Nova 6000 platform was used for PE250 sequencing (Guangdong Magigene Biotechnology Co., Ltd. Guangzhou, China). All raw fastq files were quality-filtered using Trimmomatic software [34]. FLASH was used to merge pair-ended sequences after barcodes and primers were removed [35]. Following this, UPARSE clustered these sequences into operational taxonomic units (OTUs) with a sequence threshold of 97% similarity, and representative OTU sequences were concurrently selected [36]. The singletons and chimeras were filtered during the UPARSE procedure.

### 2.3. Statistical Analysis

Taxonomic richness and Shannon diversity were calculated in the R 4.0.5 statistical environment (R Core Team, 2013, http://www.R-project.org/, accessed on 22 May 2020) with a rarefaction depth of 30,815 per sample (Figure S2). The fungal beta diversity was examined using principal coordinate analysis (PCoA) based on Bray–Curtis distance matrices. The analysis of similarities (ANOSIM) test was used to assess whether the soil fungal communities differed between invasive and native plants. Linear discriminant analysis effect size (LEfSe) was used to analyze significant taxa at the phylum, family, and genus levels of soil fungal communities. The linear discriminant analysis (LDA) score was set at 3.0, and the *p* value was set at 0.05. Bar graphs were used to display significant LDA scores. OriginLab 2023 (OriginLab, Northampton, MA, USA) was used to generate a heatmap between predominant phylum and physicochemical variables based on the spearman correlation. Canonical correspondence analysis (CCA) was employed to examine the relationships between edaphic factors and soil fungal community by Canoco 4.5 (Microcomputer Power, Ithaca, NY, USA). Correlated variables were excluded from CCA analysis when inflation factors exceeded 10. The contributions of soil physicochemical properties to keystone taxa were determined using random forest analysis [37]. This analysis was performed using the lm and calc.relimp functions in the “relaimpo” package using R software Version 4.0.5.

To assess the links between fungal communities, we created molecular ecological networks (MENs) and visualized them with Gephi 0.9.2. The network contained only robust relationships (Spearman’s correlation coefficient >0.6 and *p* value < 0.05). To guarantee reliable correlation, OTUs that were present in more than 50% of all samples were chosen. The network topological features (i.e., node number, edge number, average degree, average clustering coefficient, and network modularity) were calculated by “igraph” R package. The connectivity of each node was described within module connectivity (Zi) and module connectivity (Pi) indexes. The OTU nodes in the network were divided into four groups based on their Zi and Pi values: peripherals (Zi < 2.5, Pi < 0.62), connectors (Pi > 0.62), provincial hubs (Zi > 2.5), and kinless hubs (Zi > 2.5 and Pi > 0.62). The latter three categories were defined as keystone taxa [38]. The Zi-Pi plots based on OTUs topological features were constructed by R statistical platform.

## 3. Results

### 3.1. Fungal Diversity and Composition in A. palmeri and Native Rhizosphere Soils

**Figure 1:**
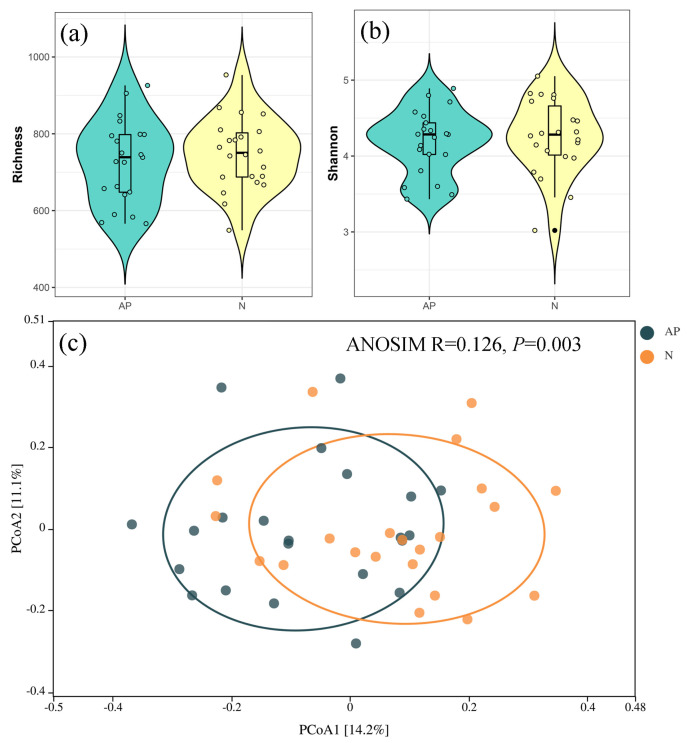
(**a**,**b**) Richness and Shannon indices of the soil fungal community and (**c**) principal coordinate analysis (PCoA) based on the Bray−Curtis distance matrix, which describes the distribution patterns of fungal communities in *A. palmeri* (AP) and native plants (N), respectively.

**Figure 2:**
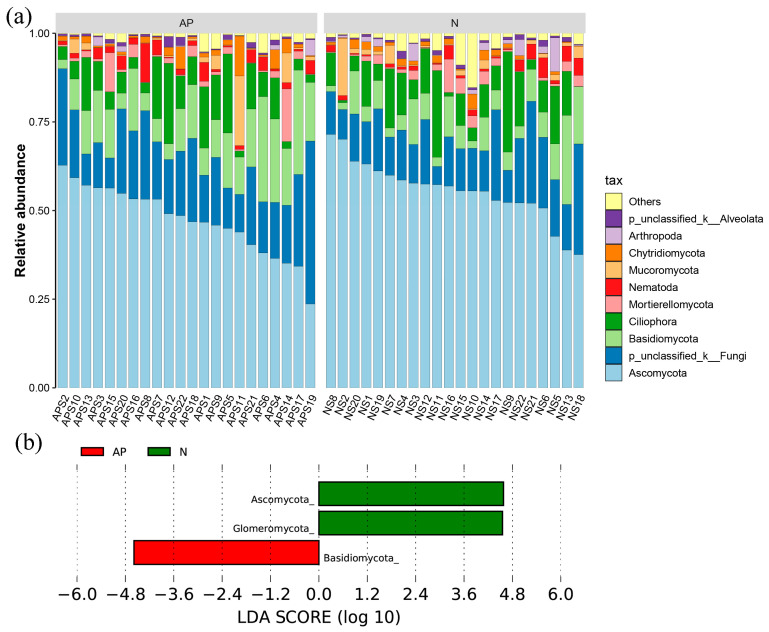
(**a**) Taxonomic composition of the soil fungal community under *A. palmeri* (AP) and native (N) rhizosphere soils across 22 sites. (**b**) Biomarkers at phylum level between *A. palmeri* (AP) and native (N) rhizosphere soils. Only LDA effect sizes > 3 are shown.

The soil fungal richness and Shannon index did not vary significantly between invasive and native plants (Figure 1a,b). The PCoA analysis and ANOSIM test (Figure 1c) showed a significant difference in the soil fungal community composition between invasive and native plants (*R* = 0.128, *p* = 0.003). The first principal component explained 14.2% of the variance, while the second principal component explained 11.1% of the variance. Ascomycota, unclassified-k-Fungi, Basidiomycota, Ciliophora, Mortierellomycota, Nematoda, Mucoromycota, Chytridiomycota, Arthropoda, and p- unclassified-k-Alveolata were the top 10 phyla with the highest relative abundance among 44 soil samples within 22 sites (Figure 2a). Linear discriminant analysis effect size (LEfSe) analysis revealed that the relative abundance of Basidiomycota was significantly greater, whereas Ascomycota and Glomeromycota were significantly lower in the rhizosphere soils of *A. palmeri* than that in native plants (Figure 2b).

### 3.2. Fungal Significant Groups Associated with Plant Invasion

**Figure 3:**
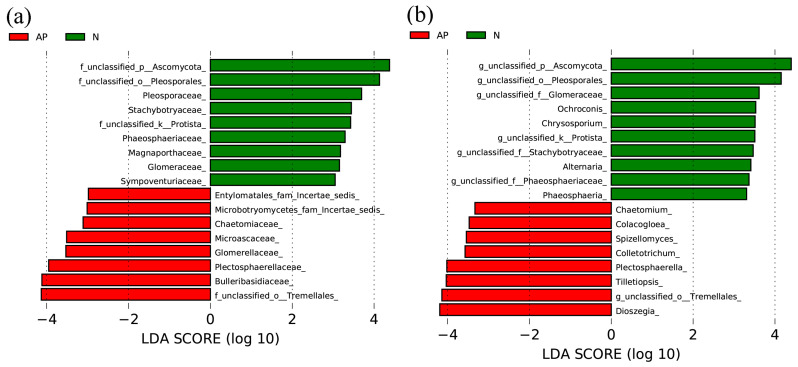
Differentially enriched fungal species at family (**a**) and genus (**b**) levels between *A. palmeri* (AP) and native (N) rhizosphere soils. Statistical significance was determined by LefSe analysis (only LDA effect sizes > 3 are shown).

LEfSe analysis showed that, at the family level, f_unclassified_o__Tremellales, Bulleribasidiaceae, Plectosphaerellaceae, Glomerellaceae, Microascaceae, Chaetomiaceae, Microbotryomycetes_fam_Incertae_sedis, and Entylomatales_fam_Incertae_sedis were significantly enriched in the rhizosphere soils of *A. palmeri*, while the abundance of f_unclassified_p__Ascomycota, f_unclassified_o__Pleosporales, Pleosporaceae, Stachybotryaceae, f_unclassified_k__Protista, Phaeosphaeriaceae, Magnaporthaceae, Glomeraceae, and Sympoventuriaceae was significantly lower than that in native rhizosphere soils (Figure 3a). At the genus level, *Dioszegia*, *g*_*unclassified*_*o*__*Tremellales*, *Tilletiopsis*, *Plectosphaerella*, *Colletotrichum*, *Spizellomyces*, *Colacogloea*, and *Chaetomium* were significantly enriched in the rhizosphere soils of *A. palmeri*, whereas the abundance of *g*_*unclassified*_*p*__*Ascomycota*, *g*_*unclassified*_*o*__*Pleosporales*, *g*_*unclassified*_*f*__*Glomeraceae*, *Ochroconis*, *Chrysosporium*, *g*_*unclassified*_*k*__*Protista*, *g*_*unclassified*_*f*__*Stachybotryaceae*, *Alternaria*, *g*_*unclassified*_*f*__*Phaeosphaeriaceae*, and *Phaeosphaeria* was significantly lower than that in the native rhizosphere soils (Figure 3b).

### 3.3. Fungal Co-Occurrence Networks and Keystone Species

**Figure 4:**
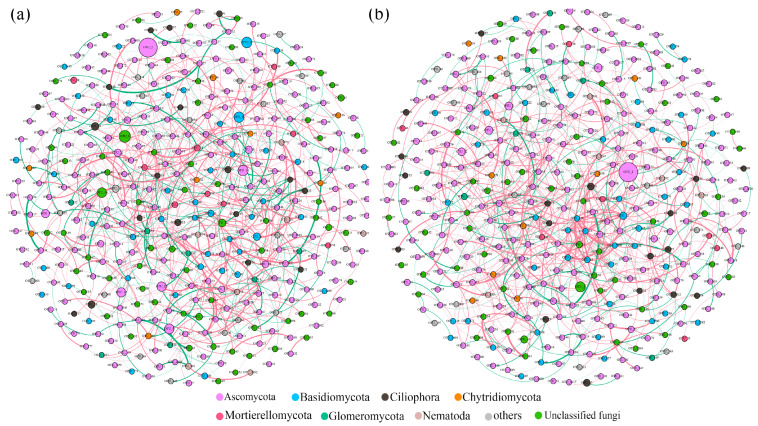
The co-occurrence patterns of the soil fungal community under invasive (**a**) and native (**b**) conditions. The size of the nodes is proportional to the relative abundance, and the color is determined depending on the level of the phylum to which they belong. The edge color represents positive (red) and negative (green) correlations. The thickness of each edge (the connection between two nodes) is proportional to the value of Spearman’s correlation coefficient.

To investigate the co-occurrence patterns of soil fungal communities, molecular ecological networks of invasive and native plants were built (Figure 4a,b). The networks of *A. palmeri* and native plants showed positive correlation ratios of 69.65% and 70.23%, respectively, demonstrating that cooperative relationships dominated in the maintenance of soil fungal interactions (Table S2). There were 401 nodes in the invaded network, which was more than the native network (389). The edge numbers of the invasive network were comparable to those of the native network (814 and 817, respectively). The invaded plots’ soil fungal network displayed a higher modularity value (0.635) (Table S2).

**Figure 5:**
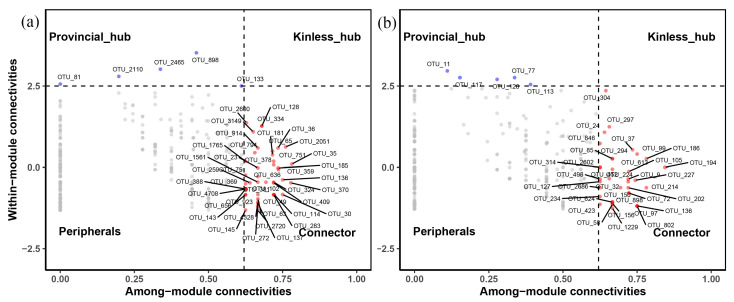
Zi−Pi plots predict keystone taxa in soil fungal communities of *A. palmeri* (**a**) and native (**b**) rhizosphere soils. Each symbol represents a node (OTU). The threshold values of Zi and Pi for categorizing OTUs were 2.5 and 0.62, respectively.

The keystone taxa of soil fungal communities were investigated further, and they were screened using the Zi-Pi value (Figure 5a,b). In addition to the peripheral hubs, the other three hubs were regarded as keystone taxa, and the difference test of keystone taxa between invasive and native plants were listed in Table S3. The majority of the nodes in the invasive and native communities were found on the peripherals, with the remainder located within the provincial hubs and connectors, whereas the two molecular ecological networks lacked kinless hubs. There were 48 keystone taxa (ranging from 0.005 to 1.429%) in the soil fungal communities of *A. palmeri*, mostly belonging to *Saitozyma*, *Alternaria*, *Colacogloea*, *Cephalotrichum*, *Myrmecridium*, *Podospora*, *Nigrospora*, *Gibellulopsis*, *Rhizopus*, *Mortierella*, *Stachybotrys*, *Bipolaris*, *Aspergillus*, *Myrothecium*, *Leptospora*, and *Phoma*, accounting for only 6.37% of the total reads. There were 41 keystone taxa (ranging from 0.006 to 2.307%) in the soil fungal communities of native plants, including *Hannaella*, *Dileptus*, *Myrmecridium*, *Alternaria*, *Periconia*, *Cyphellophora*, *Clonostachys*, *Fusariella*, *Colletotrichum*, *Mortierella*, *Beauveria*, *Lectera*, and *Phaeosphaeria*, accounting for only 9.87% of the total reads. The keystone of OTU_137 (*Colacogloea*), OTU_36, OTU_1765, OTU_370, OTU_136 (*Alternaria*), and OTU_81 in *A. palmeri* showed significant variation compared with native fungal communities. *Colacogloea* were abundant in *A. palmeri* plots, whereas *Alternaria* were dominated in native plots (Table S3).

### 3.4. Associations between Fungal Community and Edaphic Factors

**Figure 6:**
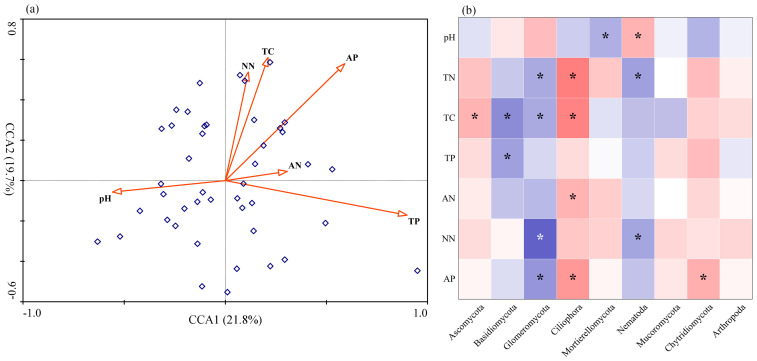
(**a**) Canonical correspondence analysis (CCA) ordination plots depicting the relationships between the fungal community and selected soil properties. (**b**) A heatmap showing the correlation between dominant phyla and physicochemical variables. Red color indicated positive correlations, and blue color indicated negative correlations. * *p* < 0.05. Abbreviations: TN, total nitrogen; TC, total carbon; TP, total phosphorus; AN, ammonium nitrogen; NN, nitrate nitrogen; and AP, available phosphorus.

CCA analysis showed that the eigenvalues of axis 1 and axis 2 were 21.8% and 19.7%, respectively, which explained 41.5% of the relationship between fungal communities and edaphic factors (Figure 6a). After excluding TN with expansion factors greater than 10, Monte Carlo results revealed that TP (*p* = 0.001, *F* = 2.04), TC (*p* = 0.002, *F* = 1.68), and AP (*p* = 0.026, *F* = 1.45) were the most significant factors affecting soil fungal community structure. The relationship between the prevalent phyla and the soil physicochemical characteristics was further examined using Spearman correlation analysis (Figure 6b). Soil total carbon was found to be significantly positively correlated with Ascomycota and Ciliophora, while negatively correlated with Basidiomycota and Glomeromycota. Soil total phosphorus and available phosphorus were significantly negatively correlated with Basidiomycota, and soil available phosphorus was significantly positively correlated with Ciliophora. According to random forest analysis, soil total carbon best explained (*p* < 0.05) the occurrence of keystone taxa among the soil factors (Figure S3).

## 4. Discussion

Our research is the first to provide tangible experimental proof of the ecological adaption strategies and response characteristics of soil fungal communities to the invasion of *A. palmeri* in northern China. The invasion of *A. palmeri* dramatically changed the soil fungal community composition, according to PCoA and ANOSIM analyses. The impacts of plant invasion on the microbial assembly have also been observed in numerous earlier investigations [8,12,16,39]. Soil physicochemical properties influence fungal community composition [40,41,42]. The CCA results demonstrated that soil total carbon (TC), total phosphorus (TP), and available phosphorus (AP) all significantly influenced the fungal community structure. Soil TC was found to be significantly linked with the relative abundance of Ascomycota, Basidiomycota, Ciliophora, and Glomeromycota, AP with Ciliophora and Glomeromycota, and TP with Basidiomycota. Hence, soil properties may largely affect the abundance of these four phyla in order to alter the assembly of the fungal community [42]. The influence mechanism of edaphic variables on the soil fungal community needs to be further studied in order to comprehend the changes in the soil fungal community and predict its functional consequences in response to *A. palmeri* invasion. In contrast to native plants, *A. palmeri* had a much higher abundance of Basidiomycota and a noticeably reduced abundance of Ascomycota in its rhizosphere soil. Basidiomycota played an important role in the degradation of lignin-rich plant litter [43]. Regardless of ecological strategies, Basidiomycota was better adapted than Ascomycota to resource allocation and spatial exploration in varied habitats, according to Bödeker [44]. Glomeromycota, a kind of representative arbuscular mycorrhizal fungi (AMF), was detected in lower relative abundance in the invaded plots, indicating that the development of invasive plants by symbiosis with fast-growing AMF may not be as important for *A. palmeri* in these particular habitats as other researchers have suggested [8,12]. It was possible that changes in biomass allocation were the cause of the large Glomeromycota differential between invaded and native habitats. AMF preferred native plants that were more adapted to the local environment [12,45]. Additionally, *A. palmeri* is an annual plant; therefore, it may be less dependent on the AMF reciprocal interaction and less reliant on soil reciprocal organisms, which could speed up the development of its population after interference [12,46,47]. The considerable changes in the abundance of Ascomycota, Basidiomycota, and Glomeromycota may be indicative of significant changes in the ecosystem’s subterranean processes related to *A. palmeri* invasion.

The use of LefSe analysis to identify biomarkers in microbial communities is a reliable method. The results revealed that *Dioszegia*, *Tilletiopsis*, *Plectosphaerella*, *Colletotrichum*, *Spizellomyces*, *Colacogloea*, and *Chaetomium* were biomarkers in the rhizosphere soil of *A. palmeri*, and *Ochroconis*, *Chrysosporium*, *Alternaria*, and *Phaeosphaeria* were regarded as biomarkers in the rhizosphere soil of native plants. Different biomarkers were found in invasive and native soil communities, which could be related to the different ecological strategies of *A. palmeri* and native vegetations. *Dioszegia* was identified as a keystone taxon in the agroecosystem by Banerjee [48]. *Tilletiopsis* species may produce antifungal compounds and hydrolytic enzymes that act as antagonists of pathogens and indirectly promote plant growth [49]. Although most *Colletotrichum* species were destructive pathogens [50], some members such as *Colletotrichum tofieldiae* colonized in Arabidopsis roots and transferred phosphorus to the host under phosphate deficiency conditions to promote plant growth [51], and *Colletotrichum siamense* had growth-promoting effects and suppressed *Fusarium oxysporum* symptoms in tomato plants [52]. A saprophytic yeast known as *Colacogloea* was highly competitive, tolerant of harsh environments, and non-pathogenic to humans, animals, or plants [53]. *Spizellomyces* may have more extensive pathways that help metabolize nutrients that were typically hard to obtain, boosting adaptability to a variety of environments [54]. Members of the Chaetomiaceae family are well-known for producing cellulase and hemicellulase [55]. Previous research found Chaetomium members to be potential antagonists of many soil-borne pathogens as well as beneficial fungal groups in plants [56,57,58]. The pathogens of *Alternaria* and *Phaeosphaeria* were enriched in the rhizosphere soil of native plants. *Alternaria* is a widespread pathogen that is responsible for 20–80% of agricultural losses in field crops, horticultural crops, planted crops, and forest plants [59,60]. *Phaeosphaeria* devastated important commercial grasses and cereals [61,62]. The more prevalent antagonists (*Chaetomium* and *Tilletiopsis*) in the rhizosphere of *A. palmeri* may explain the significant decline in *Alternaria* and *Phaeosphaeria*. The findings suggested that these taxa as biomarkers may play an important role in soils invaded by *A. palmeri* and might be adapted to soil niches that are favorable for plant invasion.

The co-occurrence network reflected potential microbial interactions. The proportion of positive links in invasive and native networks was 69.65% and 70.23%, respectively, indicating that mutualism or commensalism may have had a substantial influence on how the community was shaped [63]. In comparison to native plants, the invasion of *A. palmeri* increased the negative relationship between the soil fungal community, implying that *A. palmeri* fungal community faced higher resource competition [64,65]. The lower edges, average degree, and clustering coefficient showed that the *A. palmeri* invasion reduced the complexity of the soil fungal network [66]. The invasive soils’ greater modularity values indicated a more stable microbial community [8,67,68]. The redistribution of nutrients and the stability of ecosystem functions were facilitated by a more stable co-occurrence network [38]. *A. palmeri* had an average path length that was shorter than native plants, indicating that the network was more effective at transporting mass or nutrients [69]. We concluded that *A. palmeri* invasion may form a simpler but more effective network than native plants, affecting soil fertility and plant productivity.

Changes in the topological properties of microbial networks coincided with the replacement of keystone taxa. Keystone OTUs were placed in the most central and well-connected areas of the network [70]. Through frequent interactions with other members, keystone taxa played an important role in maintaining network stability, and their removal may destabilize modules or networks [21,71]. To the best of our knowledge, this study is the first attempt to link changes in keystone taxa of the soil fungal community with the invasion of *A. palmeri*. According to our findings, the keystone taxa of invasive and native plants were noticeably different. The majority of the keystone taxa identified in this study belonged to phyla of Ascomycota, Basidiomycota, and Mortierellomycota. Within the Ascomycota, *Cephalotrichum* is a saprophytic soil fungus [72]; *Myrmecridium*, a plant endophytes taxon, has been reported to produce extracellular hydrolytic enzymes and cellulase [73]; *Podospora* are well-known cellulose degraders [74]; *Nigrospora* has the capacity to improve the withstand stress of host plants and produce antifungal compounds [75]; and *Gibellulopsis* is linked to carbohydrate content [76]. Within the Basidiomycota, members of *Saitozyma* are involved in the decomposition of dead plant biomass [77]; *Colacogloea* is a saprophytic yeast that can persist in difficult conditions [53]. Within the Mortierellomycota, *Mortierella* is mostly composed of saprophytic species that perform several functions, including the degradation of cellulose and lignin [78]. When the relative abundance of keystone taxa was compared between invaded and native plots, *Colacogloea* increased dramatically, while *Alternaria* decreased significantly in the rhizosphere soil of *A. palmeri*. This result agreed with the indicator taxa determined by LEfSe analysis, indicating that using these methods to identify the keystone taxa in our study was valid [15]. The keystone taxa in the invasive plots included a variety of saprophytic fungi, such as Mortierellomycota (e.g., genus: *Mortierella*) and yeast (e.g., genus: *Colacogloea* and *Saitozyma*), which may result in different soil organic carbon contents in the invasive and native plants because saprophytic fungi decompose more quickly [40]. We tentatively suspect that saprophytic fungi are crucial in regulating *A. palmeri* invasion. In addition, our random forest analysis revealed that soil total carbon was a determinant of keystone taxa. Keystone taxa may influence the organic carbon content of soil, thereby influencing the soil total carbon content. In conclusion, we believe that changes in keystone species and members may be one of the primary strategies for *A. palmeri* invasion and that this should be confirmed in the future.

## 5. Conclusions

In conclusion, our research revealed that the soil fungal community, rather than alpha diversity, was significantly altered in response to *A. palmeri* invasion. Soil total phosphorus, total carbon, and available phosphorus were the main determinants of fungal community structure. The relative abundance of Basidiomycota increased while Ascomycota and Glomeromycota decreased after plant invasion. More antagonists (e.g., *Chaetomium* and *Tilletiopsis*) found in invaded soils may inhibit pathogenic fungi (e.g., *Alternaria* and *Phaeosphaeria*). Compared to native plants, *A. palmeri* created a simpler but more stable soil fungal network. Moreover, the replacement of keystone taxa may be associated with the invasion of *A. palmeri*. Our findings provide insight into the composition and co-occurrence patterns of fungal communities in *A. palmeri*-invaded ecosystems.

## Figures and Tables

**Table 1:** Ecological characteristics of the 22 sampling sites.

Site	Province	Longitude (°)	Latitude (°)	Mean Annual Temperature	Mean Annual Precipitation	Habitat Types
S1	Tianjin	116.92	38.73	506	12.96	Wasteland neighbouring roadside
S2	Tianjin	117.02	38.90	515	12.92	Wasteland neighbouring roadside
S3	Tianjin	116.96	39.04	520	12.84	Wasteland
S4	Tianjin	117.16	39.15	566	13.38	Wasteland
S5	Tianjin	117.28	39.64	549	11.95	Wasteland neighbouring roadside
S6	Tianjin	117.37	39.44	554	12.13	Wasteland
S7	Tianjin	117.51	39.15	551	12.43	Wasteland neighbouring roadside
S8	Tianjin	117.42	38.96	555	13.03	Wasteland neighbouring roadside
S9	Tianjin	116.93	39.39	535	12.39	Wasteland neighbouring roadside
S10	Tianjin	116.95	39.16	532	12.69	Wasteland neighbouring roadside
S11	Tianjin	117.39	40.03	564	11.73	Wasteland neighbouring roadside
S12	Tianjin	117.85	39.48	588	11.79	Wasteland neighbouring roadside
S13	Hebei	117.06	39.66	543	11.85	Wasteland neighbouring roadside
S14	Hebei	116.92	39.88	538	11.68	Wasteland neighbouring roadside
S15	Hebei	116.71	39.60	546	12.16	Wasteland
S16	Beijing	116.77	39.71	545	11.92	Wasteland
S17	Beijing	116.46	39.80	570	12.31	Wasteland
S18	Beijing	116.35	39.82	571	12.40	Wasteland
S19	Hebei	116.75	39.32	535	12.39	Wasteland neighbouring roadside
S20	Hebei	116.42	39.41	556	12.21	Wasteland
S21	Hebei	116.76	39.15	530	12.62	Wasteland neighbouring roadside
S22	Hebei	116.45	39.09	512	12.67	Wasteland neighbouring roadside

## Data Availability

Raw FASTQ files and assembled sequences have been deposited in the NCBI under the BioProject PRJNA947070.

## Supplementary Materials

The following supporting information can be downloaded at: https://www.mdpi.com/article/10.3390/jof9050509/s1. Figure S1: Main soil factors affecting keystone taxa characterized by random forest modeling analysis. Figure S2: The rarefaction curve of 22 samples. Figure S3: Main soil factors affecting keystone taxa characterized by random forest modeling analysis. Table S1: Major topological properties of fungal networks in *A. palmeri* (AP) and native (N) rhizosphere soils. Table S2: Relative abundance and difference test of keystone taxa in *A. palmeri* (AP) and native(N) rhizosphere soils. ** *p* < 0.01, * *p* < 0.05. Table S3: Relative abundance and difference test of keystone taxa in A. palmeri (AP) and native(N) rhizosphere soils. ** *p* < 0.01, * *p* < 0.05.
