# Supplementary material for: Responses of Fungal Assembly and Co-Occurrence Network of Rhizosphere Soil to Amaranthus palmeri Invasion in Northern China
Source: J Fungi (Basel). 2023 Apr 25;9(5):509. doi: 10.3390/jof9050509 (PMC10219470; doi:10.3390/jof9050509)
Supplement: Supplementary file 1 [file jof-09-00509-s001.zip › jof-2328995-supplementary.pdf]

Responses of Fungal Assembly and Co-Occurrence Network of Rhizosphere Soil to *Amaranthus palmeri* Invasion in Northern China

Mei Zhang [1], Kefan Wang [1], Cong Shi [2], Xueying Li [1], Zhenlu Qiu [1] and Fuchen Shi [1,*]

[1] Department of Plant Biology and Ecology, College of Life Sciences, Nankai University, Tianjin 300071, China
[2] School of Environmental Science and Engineering, Tiangong University, Tianjin 300387, China
[*] Correspondence: fcshi@nankai.edu.cn; Tel.: +86-22-23502477

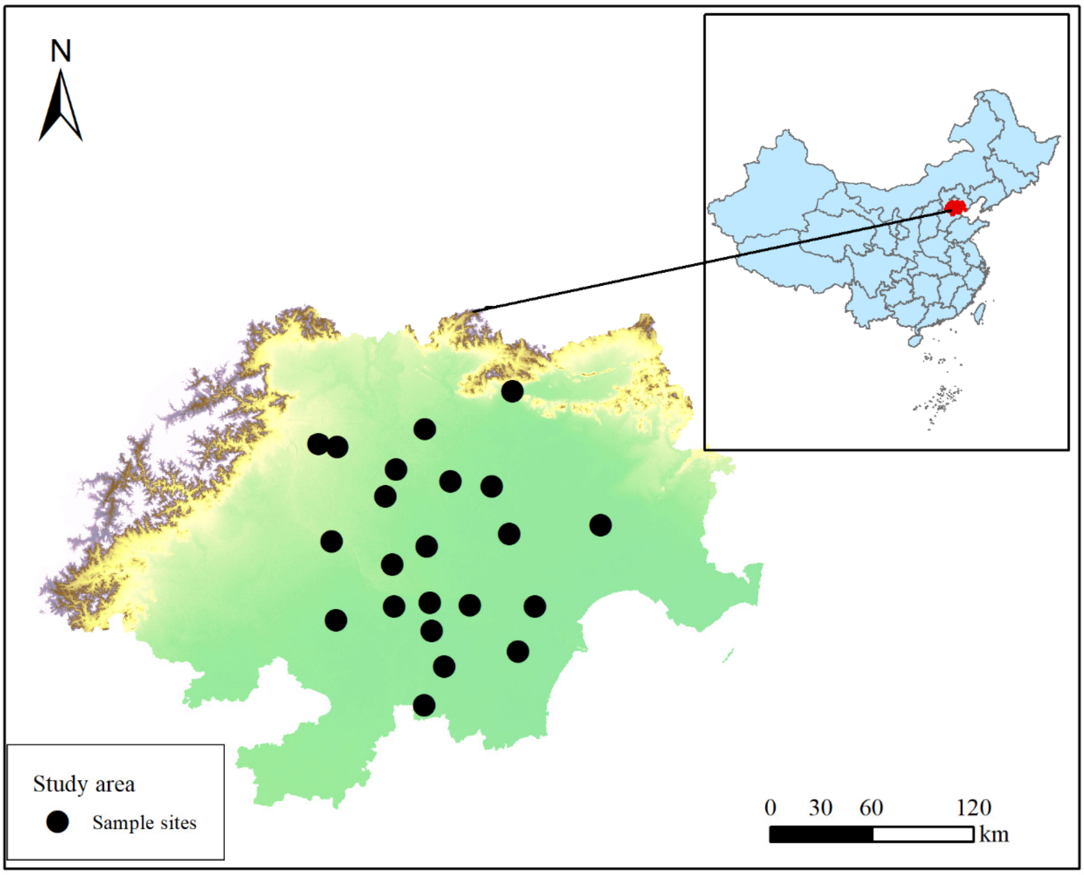

Figure S1. Location of the study area and sample sites.

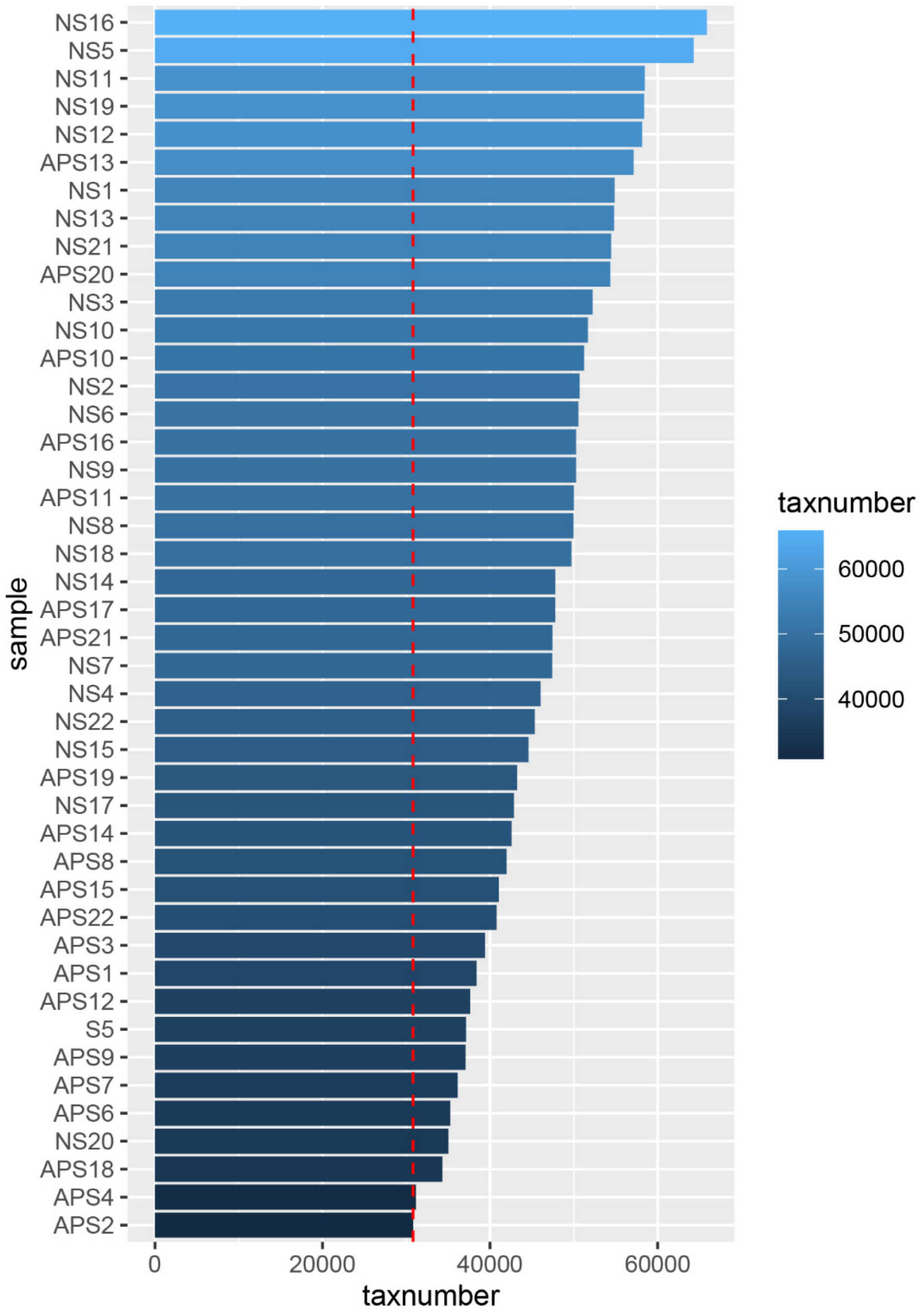

Figure S2. The rarefaction curve of 22 samples.

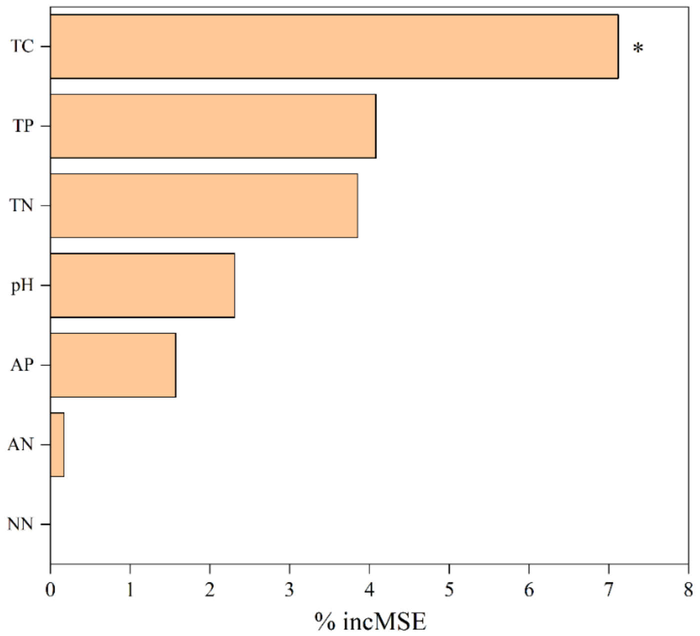

Figure S3. Main soil factors affecting keystone taxa characterized by random forest modeling analysis. The figure shows the random forest analysis results with primary predictor importance (i.e., the percent increase in mean square error (%IncMSE). * $p < 0.05$. Abbreviation: TN, total nitrogen; TC, total carbon; TP, total phosphorus; AN, ammonium nitrogen; NN, nitrate nitrogen; AP, available phosphorus.

Table S1 Vegetation characteristics of the 22 sampling sites.

**Site**	**Province**	**Dominant accompanying species in invasive patches**	**Dominant species in native patches**
1	Tianjin	*Chloris virgata, Salsola collina*	*Phragmites australis, Digitaria sanguinalis,Chloris virgata, Artemisia lavandulifolia, Chenopodium glaucum*
2	Tianjin	*Setaria viridis, Chenopodium album*	*Setaria viridis, Suaeda glauca*
3	Tianjin	*Amaranthus viridis*	*Setaria viridis, Amaranthus viridis, Abutilon theophrasti, Xanthium sibiricum*
4	Tianjin	*Setaria viridis*	*Setaria viridis, Digitaria sanguinalis, Amaranthus viridis, Chenopodium album*
5	Tianjin	*Portulaca oleracea*	*Setaria viridis, Artemisia lavandulifolia*
6	Tianjin	*Portulaca oleracea*	*Setaria viridis, Chenopodium album, Suaeda glauca, Portulaca oleracea*
7	Tianjin	*Setaria viridis*	*Amaranthus viridis, Xanthium strumarium*
8	Tianjin	*Portulaca oleracea*	*Chenopodium album, Setaria viridis*
9	Tianjin	*Setaria viridis*	*Kochia scoparia, Setaria viridis*
10	Tianjin	*Chloris virgata*	*Artemisia scoparia, Kochia scoparia, Setaria viridis, Digitaria sanguinalis,Chloris virgata*
11	Tianjin	*Digitaria sanguinalis*	*Kochia scoparia, Setaria viridis, Digitaria sanguinalis,Chloris virgata*
12	Tianjin	*Eleusine indica*	*Kochia scoparia, Echinochloa crusgalli , Digitaria sanguinalis,Eleusine indica, Chloris virgata*
13	Hebei	*Chenopodium album*	*Abutilon theophrasti, Chenopodium album, Digitaria sanguinalis*
14	Hebei	*Setaria viridis*	*Amaranthus viridis, Setaria viridis, Kochia scoparia*

15	Hebei	*Eleusine indica*	*Xanthium strumarium, Eleusine indica*
16	Beijing	*Setaria viridis*	*Xanthium strumarium, Setaria viridis, Eleusine indica*
17	Beijing	*Kochia scoparia*	*Kochia scoparia, Setaria viridis*
18	Beijing	*Setaria viridis*	*Chenopodium album, Setaria viridis, Abutilon theophrasti*
19	Hebei	*Eleusine indica*	*Xanthium strumarium, Eleusine indica, Setaria viridis*
20	Hebei	*Chenopodium album*	*Chenopodium album, Digitaria sanguinalis, Abutilon theophrasti, Setaria viridis*
21	Hebei	*Amaranthus viridis*	*Setaria viridis, Eleusine indica, Digitaria sanguinalis, Amaranthus viridis*
22	Hebei	*Eleusine indica*	*Eleusine indica, Chloris virgata, Echinochloa crusgalli*

Table S2 Major topological properties of fungal networks in *A. palmeri* (AP) and native (N) rhizosphere soils.

network	AP	N
node number	401	389
edge number	814	817
net average degree	4.06	4.20
net positive	567	574
net negative	247	243
edge density	0.0101	0.0108
net diameter	12	12
clustering coefficient	0.180	0.216
Average path length	4.89	4.93
Assortativity	0.2537	0.3897
net degree centrality	0.0273	0.0329
average neighbors	5.34	5.53
net modularity	0.635	0.619
HarMonicGeoDis	10.69	9.10
r2.power.law	0.727	0.814

Table S3 Relative abundance and difference test of keystone taxa in *A. palmeri* (AP) and native(N) rhizosphere soils. **$p < 0.01$, * $p < 0.05$.

Invaded	Genus	Family	Phyla	AP	N	*P*
OTU_102	——	——	Ascomycota	0.001152	0.001572	ns
OTU_114	*Saitozyma*	Trimorphomycetaceae	Basidiomycota	0.001484	0.001466	ns
OTU_128	*Mortierella*	Mortierellaceae	Mortierellomycota	0.002440	0.002577	ns
OTU_133	——	——	Ascomycota	0.006608	0.002450	ns
**OTU_136**	***Alternaria***	**Mortierellaceae**	**Ascomycota**	**0.000615b**	**0.001898a**	**
**OTU_137**	***Colacogloea***	**Microbotryomycetes**	**Basidiomycota**	**0.002031a**	**0.000845b**	*
OTU_143	*Cephalotrichum*	Microascaceae	Ascomycota	0.004725	0.001975	ns
OTU_145	——	——	Ciliophora	0.000127	0.000103	ns
OTU_1561	——	Glomeraceae	Glomeromycota	0.000055	0.000127	ns
**OTU_1765**	**——**	**——**	**Ascomycota**	**0.001376a**	**0.000727b**	*
OTU_181	——	——	k:Fungi	0.001151	0.000996	ns
OTU_185	unidentified	unidentified	Ascomycota	0.000395	0.001764	ns
OTU_2051	——	——	Ascomycota	0.000245	0.000417	ns
OTU_2110	*Myrmecridium*	Myrmecridiaceae	Ascomycota	0.000214	0.000189	ns
OTU_23	*unidentified*	unidentified	Ascomycota	0.006816	0.007427	ns
OTU_2465	——	unidentified	k:Fungi	0.001022	0.000836	ns
OTU_259	——	unidentified	k:Alveolata	0.000645	0.000313	ns
OTU_2600	——	unidentified	Basidiomycota	0.001235	0.000354	ns
OTU_272	*Podospora*	Lasiosphaeriaceae	Ascomycota	0.000308	0.000978	ns
OTU_2720	*Nigrospora*	Trichosphaeriaceae	Ascomycota	0.000117	0.000108	ns
OTU_283	*Gibellulopsis*	Plectosphaerellaceae	Ascomycota	0.000502	0.000468	ns
OTU_30	*Rhizopus*	Rhizopodaceae	Mucoromycetes	0.011660	0.001876	ns
OTU_3149	*Stachybotrys*	Stachybotryaceae	Ascomycota	0.000240	0.000754	ns
OTU_324	*unidentified*	Stachybotryaceae	Ascomycota	0.000155	0.000574	ns
OTU_334	——	——	Fungi	0.001252	0.000438	ns
OTU_35	——	——	Ciliophora	0.006566	0.004583	ns
OTU_359	——	——	Ascomycota	0.000625	0.000513	ns
**OTU_36**	**——**	**——**	**Fungi**	**0.014287a**	**0.000219b**	***
OTU_369	——	——	Fungi	0.000090	0.000599	ns
**OTU_370**	**——**	**——**	**Fungi**	**0.000480a**	**0.000336b**	*
OTU_378	*Mortierella*	Mortierellaceae	Mortierellomycota	0.000656	0.000807	ns
OTU_381	——	——	Alveolata	0.000702	0.000530	ns
OTU_388	——	Chaetomiaceae	Ascomycota	0.000453	0.000822	ns
OTU_409	——	Spathidiidae	Ciliophora	0.000313	0.000559	ns
OTU_4328	*Bipolaris*	Pleosporaceae	Ascomycota	0.000183	0.000140	ns
OTU_4708	*Aspergillus*	Aspergillaceae	Ascomycota	0.000159	0.000235	ns
OTU_49	——	——	Fungi	0.004587	0.001028	ns
OTU_63	——	Trichocomaceae	Ascomycota	0.004788	0.000161	ns

OTU_636	unidentified	Cordycipitaceae	Ascomycota	0.000134	0.000220	ns
OTU_65	——	——	Fungi	0.001598	0.003791	ns
OTU_656	*Myrothecium*	Stachybotryaceae	Ascomycota	0.000432	0.000336	ns
OTU_75	——	——	k:Alveolata,p:Ciliophora	0.000946	0.004816	ns
OTU_751	*Leptospora*	Phaeosphaeriaceae	Ascomycota	0.000167	0.000186	ns
OTU_794	*Phoma*	Didymellaceae	Ascomycota	0.000133	0.000155	ns
**OTU_81**	**——**	**——**	**Ascomycota**	**0.001376b**	**0.005721a**	*
OTU_898	——	——	Ascomycota	0.001968	0.002811	ns
OTU_914	——	Glomeraceae	Ascomycota	0.000170	0.000310	ns
OTU_923	——	——	Ascomycota	0.000090	0.000060	ns
**Native**	**Genus**	**Family**	**Phyla**	**AP**	**N**	**P**
OTU_105	——	——	Basidiomycota	0.000083	0.003607	ns
OTU_11	*Hannaella*	Bulleribasidiaceae	Basidiomycota	0.013059	0.016335	ns
OTU_113	——	unidentified	k:Protista	0.000482	0.002106	ns
OTU_117	——	Nectriaceae	Ascomycota	0.001353	0.002353	ns
OTU_1229	——	Lasiosphaeriaceae	Ascomycota	0.000046	0.000232	ns
OTU_126	*Dileptus*	Dileptidae	k:Alveolata,p:Ciliophora	0.001332	0.001913	ns
OTU_127	*Myrmecridium*	Myrmecridiaceae	Ascomycota	0.001164	0.001391	ns
**OTU_136**	***Alternaria***	**Pleosporaceae**	**Ascomycota**	**0.000615b**	**0.001898a**	**
OTU_150	——	——	Ascomycota	0.001009	0.001536	ns
OTU_156	——	——	Ascomycota	0.001416	0.000586	ns
OTU_186	*Periconia*	Periconiaceae	Ascomycota	0.000866	0.001397	ns
OTU_194	——	Cordycipitaceae	Ascomycota	0.001922	0.001456	ns
OTU_202	——	——	Ascomycota	0.000372	0.001005	ns
OTU_214	——	unidentified	Ascomycota	0.000237	0.000996	ns
OTU_224	——	——	Ascomycota	0.000367	0.000524	ns
OTU_227	*Cyphellophora*	Cyphellophoraceae	Ascomycota	0.000419	0.000658	ns
OTU_234	——	unidentified	Ascomycota	0.000177	0.000223	ns
OTU_24	——	——	k:Fungi	0.009560	0.008542	ns
OTU_2602	*Clonostachys*	Bionectriaceae	Ascomycota	0.000258	0.000394	ns
**OTU_2686**	**——**	**——**	**Ascomycota**	**0.001373b**	**0.003557a**	*
OTU_294	——	unidentified	k:Fungi	0.000454	0.000490	ns
OTU_297	*Fusariella*	Hypocreales	Ascomycota	0.000108	0.000720	ns
OTU_304	——	Clavicipitaceae	Ascomycota	0.000288	0.000176	ns
OTU_314	——	Chaetomiaceae	Ascomycota	0.000587	0.000190	ns
OTU_32	——	unidentified	Ascomycota	0.003080	0.009098	ns
OTU_37	——	k:Fungi	k:Fungi	0.005712	0.002924	ns
**OTU_423**	***Colletotrichum***	**Glomerellaceae**	**Ascomycota**	**0.000355a**	**0.000311b**	*
OTU_452	——	Helotiaceae	Ascomycota	0.000119	0.000353	ns
**OTU_498**	**——**	**——**	**k:Fungi**	**0.002640a**	**0.000116b**	*
OTU_58	——	——	Mortierellomycota	0.006017	0.000249	ns
OTU_613	——	——	Ascomycota	0.000243	0.000550	ns
OTU_624	——	Dictyosporiaceae	Ascomycota	0.000096	0.000218	ns

OTU_72	——	——	Basidiomycota	0.001909	0.002146	ns
OTU_77	*Mortierella*	Mortierellaceae	Mortierellomycota	0.003890	0.003225	ns
OTU_802	*Beauveria*	Cordycipitaceae	Ascomycota	0.000301	0.000058	ns
OTU_846	——	Lasiosphaeriaceae	Ascomycota	0.000130	0.000239	ns
OTU_85	——	——	k:Fungi	0.001972	0.001940	ns
OTU_898	——	——	Ascomycota	0.001968	0.002811	ns
OTU_9	*Lectera*	Plectosphaerellaceae	Ascomycota	0.017946	0.023066	ns
OTU_97	*Phaeosphaeria*	Phaeosphaeriaceae	Ascomycota	0.001640	0.001946	ns
OTU_99	——	——	Ascomycota	0.002698	0.003053	ns
